# Acetylation dependent functions of Rab22a-NeoF1 Fusion Protein in Osteosarcoma

**DOI:** 10.7150/thno.46082

**Published:** 2020-06-19

**Authors:** Xiaoting Liang, Xin Wang, Yaohui He, Yuanzhong Wu, Li Zhong, Wen Liu, Dan Liao, Tiebang Kang

**Affiliations:** 1 Sun Yat-sen University Cancer Center, State Key Laboratory of Oncology in South China, Collaborative Innovation Center for Cancer Medicine, Guangzhou 510060, China.; 2 Fujian Provincial Key Laboratory of Innovative Drug Target Research, School of Pharmaceutical Sciences, Xiamen University, Xiang'an South Road, Xiamen, Fujian 361102, China; State Key Laboratory of Cellular Stress Biology, Xiamen University, Xiang'an South Road, Xiamen, Fujian 361102, China.

**Keywords:** osteosarcoma, Rab22a-NeoF1, acetylation, metastasis, p300/CBP

## Abstract

**Background:** Rab22a-NeoF1 fusion gene containing the 1-38aa of Rab22a (Rab22a^1-38^) plays a decisive role in driving tumor metastasis by activating RhoA via binding to SmgGDS607. However, its intercellular regulation remains unknown.

**Methods:** The Lys7 (K7) acetylation of Rab22a-NeoF1 was initially identified by mass spectrum. Co-transfection, immunoprecipitation and Western blotting were used to characterize the acetyltransferases and deacetylases responsible for the K7 acetylation of Rab22a-NeoF1, and to define the interaction of proteins. The specificity of K7 acetylation of Rab22a-NeoF1 was determined by its specific anti-K7ac-Rab22a-NeoF1 antibody and its K7R mutant. RhoA-GTP was measured by RhoA activation assay. The migration and invasion were assessed by Transwell assay without and with Matrigel matrix, respectively. The orthotopic osteosarcoma metastasis model *in vivo* was used to monitor the lung metastases of U2OS/MTX300-Luc stably expressing Vector, Rab22a-NeoF1 or its K7R mutant with or without C646, a relatively specific inhibitor of p300/CBP. The unpaired Student *t* test was used for the statistical significance.

**Results:** The K7 of Rab22a-NeoF1 is acetylated by p300/CBP while is de-acetylated by both HDAC6 and SIRT1. The K7R mutant of Rab22a-NeoF1 lacks its binding to SmgGDS607 and subsequently lost its promoting functions, such as activation of RhoA, cell migration, invasion and lung metastasis in osteosarcoma *in vitro* and *in viv*o, which are also diminished by p300/CBP inhibitor C646.

**Conclusion:**The promoting function of Rab22a-NeoF1 is dependent on its K7 acetylation in osteosarcoma, and targeting this acetylation (e.g., C646) may benefit cancer patients, in particular osteosarcoma patients, who are positive for the Rab22a^1-38^.

## Introduction

Osteosarcoma is one of the most common malignant primary cancers of bone originating from mesenchymal tissue, with relatively high incidence rates in children and adolescents 1. Patients with non-metastatic disease have 60-70% chance of 5 years survival after diagnosis; however, this chance will drop to less than 20% in patients with metastases. Exploring the clinical, epidemiological, genetic and biological aspects of osteosarcoma can enhance our understanding of the biology of osteosarcoma, thus leading to the identification of novel therapeutic strategies 2-6. Redundancy in growth signals and high heterogeneity lead to no effective molecular therapeutic target in osteosarcoma 7. Our group has just identified a fusion gene termed Rab22a-NeoF1, which is a driver for osteosarcoma lung metastasis and may be potential target for patients with osteosarcoma metastases 8.

Rab22a-NeoF1 fusion protein has 186aa comprising the 1-38aa of Rab22a (Rab22a^1-38^) and the 39-186aa encoded from the inverted intron of *DOK5*. The Rab22a^1-38^ plays a decisive role in driving osteosarcoma lung metastasis by activating RhoA through binding to SmgGDS607 via Arg4 (R4) and Lys7 (K7) 8. RhoA is a member of the Rho GTPase family that plays key roles in metastasis, and SmgGDS607 is an isoform of SmgGDS (PAR1GDS1), which is a specific GEF protein for RhoA/RhoC. However, the intercellular regulation of Rab22a-NeoF1 on RhoA remains to be explored. According to the full-length Rab22a protein model 9, K7 is blocked by the main chain oxygen atoms of Met73, Tyr74 and Arg76, whereas R4 is surface-exposed. In Rab22a-NeoF1, however, they may both be exposed because of the truncation and possibly different folding from that of wild-type Rab22a, insulting in a much higher binding affinity of Rab22a-NeoF1 to SmgGDS607 compared to wild type Rab22a 8. Lysine residue in proteins is often modified by many post-translational modifications, such as methylation, ubiquitination and acetylation, which play an important role in protein-protein interactions, protein subcellular localization, protein stability and so on 10-18. Therefore, we wondered whether K7 of Rab22a-NeoF1 may have some modification to regulate its promoting functions, as K7 may be surface-exposed. In this study, we provide evidence that Rab22a-NeoF1 is acetylated at K7, and such an acetylation is crucial for its functions.

## Materials and Methods

### Antibodies

Antibodies used in this study were as follows: Flag (D6W5B) Rabbit mAb (CST, 8146S), HA-Tag (C29F4) Rabbit mAb (CST, 3724), anti-Acetylated-Lysine Antibody (CST, 9441s), Acetylated lysine polyclonal antibody (PAB10348, abnova), RhoA (67B9) Rabbit mAb (CST, #2117S), β-Tubulin (9F3) Rabbit mAb (CST,#2128), GAPDH (D16H11) Rabbit mAb (CST, #5174), SirT1 (D1D7) Rabbit mAb (CST, #9475) ,HDAC6(D2E5) Rabbit mAb (CST, #7558), BD Pharmingen™ Purified Mouse Anti-Human p300 (BD, 554215), CBP (D6C5) Rabbit mAb (CST, #7389). mAb RAD5-8 and hRAD5-8-v1-R5 antibody were generated as previously described 8.

The specific Anti-K7ac-Rab22a-NeoF1 antibody is designed and generated by ptm-biolab according to the following peptides table 1:

### Chemicals, enzymes and other reagents

DMSO(Sigma), Salicylate (sodium salicylate) (Selleck, S3137), sirtuins inhibitor: Nicotinamide (Vitamin B3) (NAM) (Selleck,S1899), HDAC inhibitor: Trichostatin A (TSA) (Selleck, S1045), Matrigel matrix (BD, 354234), S-protein Agarose (Millipore, 69704), Rhotekin-RBD beads (binds active Rho proteins) (Cytoskeleton, RT02-A), RhoA Pull-down Activation Assay Biochem Kit (bead pull-down format) cytoskeleton; BK036, In-fusion enzyme (synbio-tech), FastDigest incision enzyme (Thermo Scientific), T4 DNA Ligase (NEB, M0202L), Streptavidin Sepharose (GE Healthcare, 17-5113-01), D-Biotin (BBI Life Sciences, 58-85-5), 5 × HAT buffer (Millipore), Transwell chamber (BD, 353097).

### Cell lines

Human cell lines HEK293T, U2OS and 143B cells were purchased from The Global Bioresource Center (ATCC) and cultured following direction from ATCC. U2OS/MTX300, U2OS/MTX300-Luc, ZOS and ZOS-M cells were cultured in high-glucose (25 mM) DMEM medium (Gibco) containing 10% fetal bovine serum (Gibco) and 50 mg/ml penicillin/streptomycin.

### Expression plasmids

For transient transfection in HEK293T cells, cDNA encoding Vector, Rab22a-NeoF1 or its K7R and K7A mutant were individually cloned into pcDNA3.1 vector (Invitrogen). For stable expression in osteosarcoma cell lines, cDNA encoding Vector, Rab22a-NeoF1 or its K7R mutant were separately cloned into lentiviral pSIN vector.

Sirtuins (SIRT1-7) and histone deacetylase (HDAC1-11) were cloned into N-terminal HA tagged pcDNA3.1 vector (Invitrogen) by in-fusion cloning.

## Methods

### Transfection and lentivirus production

Transient transfection of plasmids was performed using Lipofectamine 2,000 according to the manufacturer's instructions (Invitrogen). To generate lentiviruses, HEK293T-packaging cells were transfected with empty vector (pSIN-puro), pSIN-Rab22a-NeoF1 or pSIN-Rab22a-NeoF1-K7R constructs together with PSA and PIG (pSIN constructs:PSA:PIG=3:2:1) using Lipofectamine 2,000. Media were changed 6-7 h after transfection, and the cell supernatant was collected after 48 h. The cleared supernatant was filtered through a 0.45 mm filter, aliquoted and stored at -80°C until to be used.

### Generation of stable cell lines

Cells were infected by lentivirus supernatant in the presence of 8 mg/ml Polybrene for 18-24 h. 48 h after infection, the cells were selected using cell medium containing 0.5 mg/ml puromycin.

### Immunoblotting and immunoprecipitation

For Western blotting, cells were lysed in RIPA lysis buffer containing protease inhibitor and phosphatase inhibitor cocktails. Lysates were cleared by centrifugation at 12,000 rpm for 20 min at 4°C. For immunoprecipitation, the lysate were first incubated with anti-Flag agarose or anti-S-protein beads overnight at 4°C and then the precipitates were washed five times with cold RIPA buffer and were eluted with 5 X SDS-PAGE. After SDS-PAGE, the proteins were transfered from the gel to the membrane. The membrane was blocked in PBST with 5% nonfat milk for 1-4 h in room temperature. Incubate the membrane with appropriate dilutions of primary antibody in antibody dilution buffer overnight at 4°C. Wash the membrane with PBST for three times. Incubate the membrane with the recommended dilution of conjugated secondary antibody in 5% nonfat milk blocking buffer at room temperature for 1 h. Wash the membrane with PBST for three times. Acquire image using darkroom development techniques for chemiluminescence, or normal image scanning methods for colorimetric detection.

### Mass spectrometric analysis

HEK293T cells transfected with SFB-tagged Rab22a-NeoF1 expression plasmids for 48 h were collected and lysed with RIPA buffer, and the cell lysates were immunoprecipitated with anti-Flag agarose beads. The Flag peptide-eluted material was resolved by 10% SDS-PAGE. The Rab22a-NeoF1 bands were excised from the gel and were subjected to tryptic digestion and mass spectrometry. Protein and its modification were identified through the database search, and peptide identifications were validated with Peptide Prophet.

### Transwell assay

The migration and invasion assays of osteosarcoma cells were performed using 24 wells Boyden chambers. FBS free osteosarcoma cells suspension (U2OS: 5×10^4^ /well, U2OS/MTX300 cells: 1×10^5^ /well) were placed on the upper layer of the cell culture insert with permeable membrane coated with (invasion) or without (migration) the Matrigel matrix. 10% FBS (migration) or 20% FBS (invasion) cell culture media were added into the lower reservoir, making sure the solution touches the membrane of the insert. The cells in the chambers are then incubation in 37°C (U2OS cells for 12 h, U2OS/MTX300 cells for 24 h). Following incubation, cells left in the upper layer of the insert are wiped off using cotton swab, cells across the inserts are fixed by dipping the insert into 4% paraformaldehyde for 15 min and stained by 1% crystal violet solution for 30 min at room temperature. The inserts are washed with 1 X PBS and leave it dry in the air. Finally, the numbers of cells cross the inserts per field of view are counted under microscope.

### Cell viability assay

U2OS, or U2OS/MTX300 cells were seeded in 96-well plates at a density of 6,000 cells per well. Cells were then treated with different concentrations of C646 (0, 2.5, 5, 10, or 20 μM) or SA (0, 1, 5, 10, or 20 μM) for the indicated times, and the cell viability was measured by MTT assay.

### *In vitro* acetylation assay

Rab22a-NeoF1-WT-SFB and Rab22a-NeoF1-K7R-SFB were purified by IP using Streptavidin Sepharose beads from HEK293T cells transfected with Rab22a-NeoF1-WT-SFB or Rab22a-NeoF1-K7R-SFB plasmids, followed by eluting with 2 mg/ml D-Biotin solution in 4°C for 6 h. Rab22a-NeoF1-WT-SFB and Rab22a-NeoF1-K7R-SFB were incubated with HA-CBP purified from HEK293T cells in HAT buffer (Millipore) in a 30°C shaking incubator for 1 h. The effect of K7 acetylation was determined using anti-K7ac-Rab22a-NeoF1 antibody by Western blotting.

### RhoA GTPase activation assay

RhoA GTPase activation assay was performed with RhoA Pull-down Activation Assay Biochem Kit (bead pull-down format) following the direction of the manufacturer's protocol.

### *In vivo* studies in mice

The study is compliant with all relevant ethical regulations regarding animal research. Animal experiments were approved by the Animal Research Committee of Sun Yat-sen University Cancer Center and performed in accordance with established guidelines. U2OS/MTX300-luc cells stably overexpressing Vector, Rab22a-NeoF1 or its K7R mutant were prepared, and nude mice were purchased from Beijing Vital River Laboratory Animal Technology. 1×10^6^ cells in PBS with 1% FBS were injected into distal femur, proximal tibia of each nude mouse (10 mice per group). After two months, lung metastases of U2OS/MTX300-luc cells were measured by *in vivo* fluorescent imaging and all mice were sacrificed and lungs with metastasis were harvested, and wet lungs were weighted and lung metastasis nodes were counted. For the treatment of C646, which was dissolved in ddH2O with 7.7% DMSO and 40% PEG300 at daily dose of 10 mg/kg for 14 days, was intraperitoneally injected into mice after the injection of U2OS/MTX300-luc cells for 3 weeks.

### Statistical analysis

All experiments were performed at least three times. Data were analyzed with GraphPad Prism v.8 (GraphPad) and SPSS statistics. Student's *t*-test was used to compare the differences between two groups. *p<0.05 was considered as statistically significant while **p<0.01, ***p<0.001, ****p<0.0001 as highly significant.

## Results

### Rab22a-NeoF1 is acetylated at lysine 7

**Figure 1:**
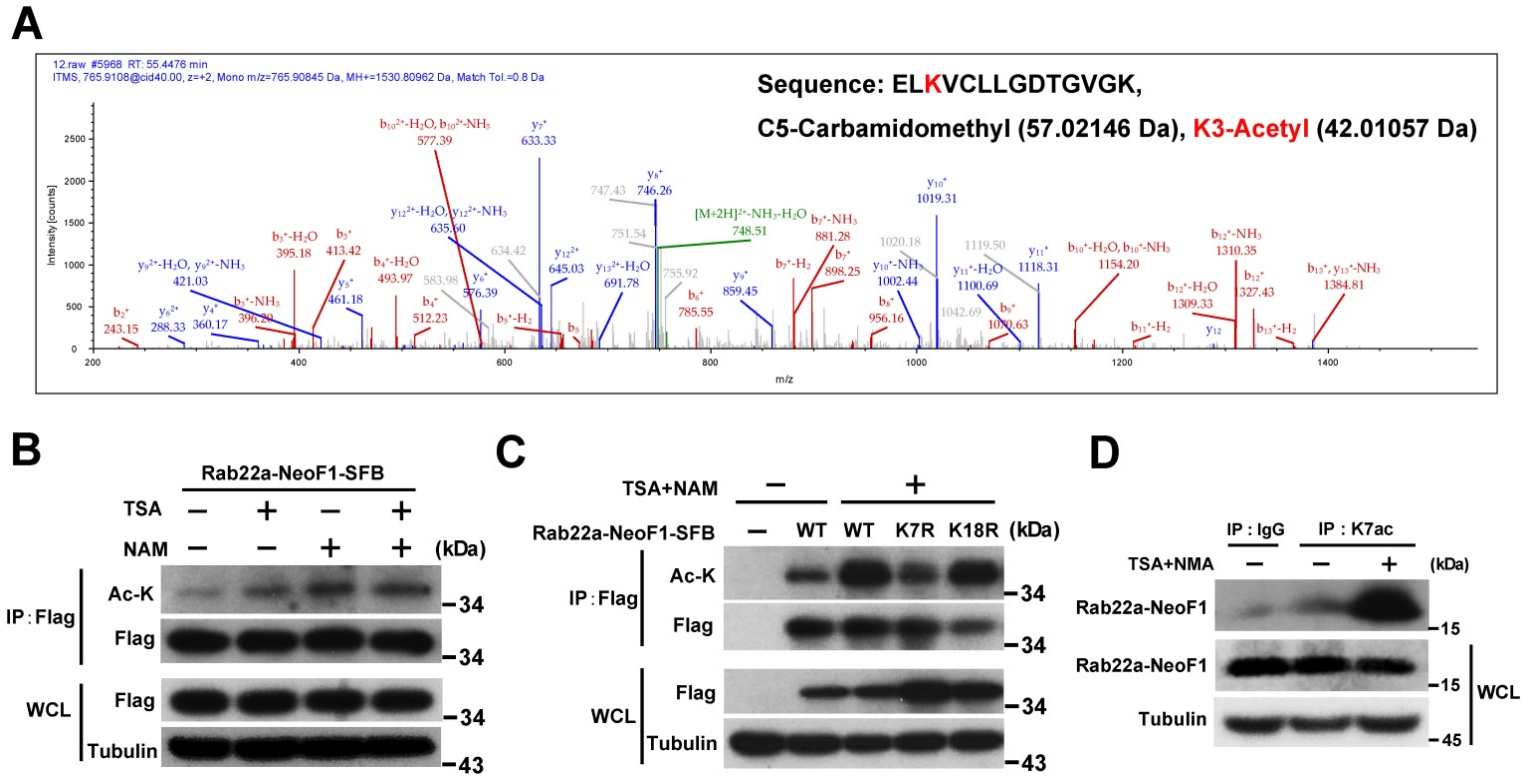
** Rab22a-NeoF1 fusion protein is acetylated at lysine 7 (K7).** (**A**) HEK293T cells transfected with Rab22a-NeoF1-SFB for 48 h, and cell lysates were subjected to immunoprecipitation (IP) with anti-Flag agarose, the IP complex was then analyzed by mass spectrometry. MS spectra of Ac-K containing the 5-ELKVCLLGDTGVGK-18 peptide obtained after trypsin digestion of the IP complex. (**B, C**) HEK293T cells transiently transfected with Rab22a-NeoF1-SFB or its K7R mutant for 24 h were treated with TSA (5 µM), NAM (5 mM), or both for 8 h. Cell lysates were subjected to IP with anti-Flag agarose, and then were analyzed by Western blotting. (**D**) ZOS-M cells were treated with both TSA (5 µM) and NAM (5 mM) for 24 h, cell lysates were subjected to IP using anti-K7ac-Rab22a-NeoF1 antibody, and then were analyzed by Western blotting using mAb RAD5-8.

In order to explore the K7 modification in Rab22a-NeoF1, we purified its SFB-tagged protein from HEK293T cells transiently expressing Rab22a-NeoF1-SFB to be subjected to mass spectrum analysis. The acetylation, ubiquitylation and di-methylation have been detected at K7 of Rab22a-NeoF1 from cells **(Figure 1A, S1A-B)**. Using mono-, di-, tri-methylation of Myc-p53 as the positive control 19-23, the di-methylation has been validated by Western blotting and was marginally altered in the mutant of Rab22a-NeoF1-K7A, in which K7 was mutated into alanine (A) (**Figure S1C-E**). The ubiquitination levels were also not changed in the mutant of Rab22a-NeoF1-K7R, in which K7 was mutated into arginine (R), even cells were treated with inhibitors of deacetylases, TSA plus NAM (**Figure S1F**). In contrast, TSA plus NAM significantly increased the lysine acetylation level of Rab22a-NeoF1 and Rab22a-NeoF1-K18R, but not Rab22a-NeoF1-K7R, as indicated by anti-acetylated lysine (anti-ac-K) antibody, whereas Rab22a-NeoF1-K18R, in which K18 was mutated into arginine (R), had no effect on its lysine acetylation level (**Figure 1B-C**). To further confirm the K7 acetylation of Rab22a-NeoF1, we generated an antibody specifically recognizing the acetylation at K7 of Rab22a-NeoF1, named as K7ac. Using this antibody, we were able to detect the K7 acetylation of endogenous Rab22a-NeoF1 in ZOS-M cells, which was dramatically increased under treatment of TSA plus NAM (**Figure 1D**). Taken together, our results determine that the majority of K7 modification in Rab22a-NeoF1 is acetylation, but not methylation or ubiquitination.

### The K7R mutant of Rab22a-NeoF1 lacks its capacities of enhancing migration, invasion and metastasis *in vitro* and *in vivo*

**Figure 2:**
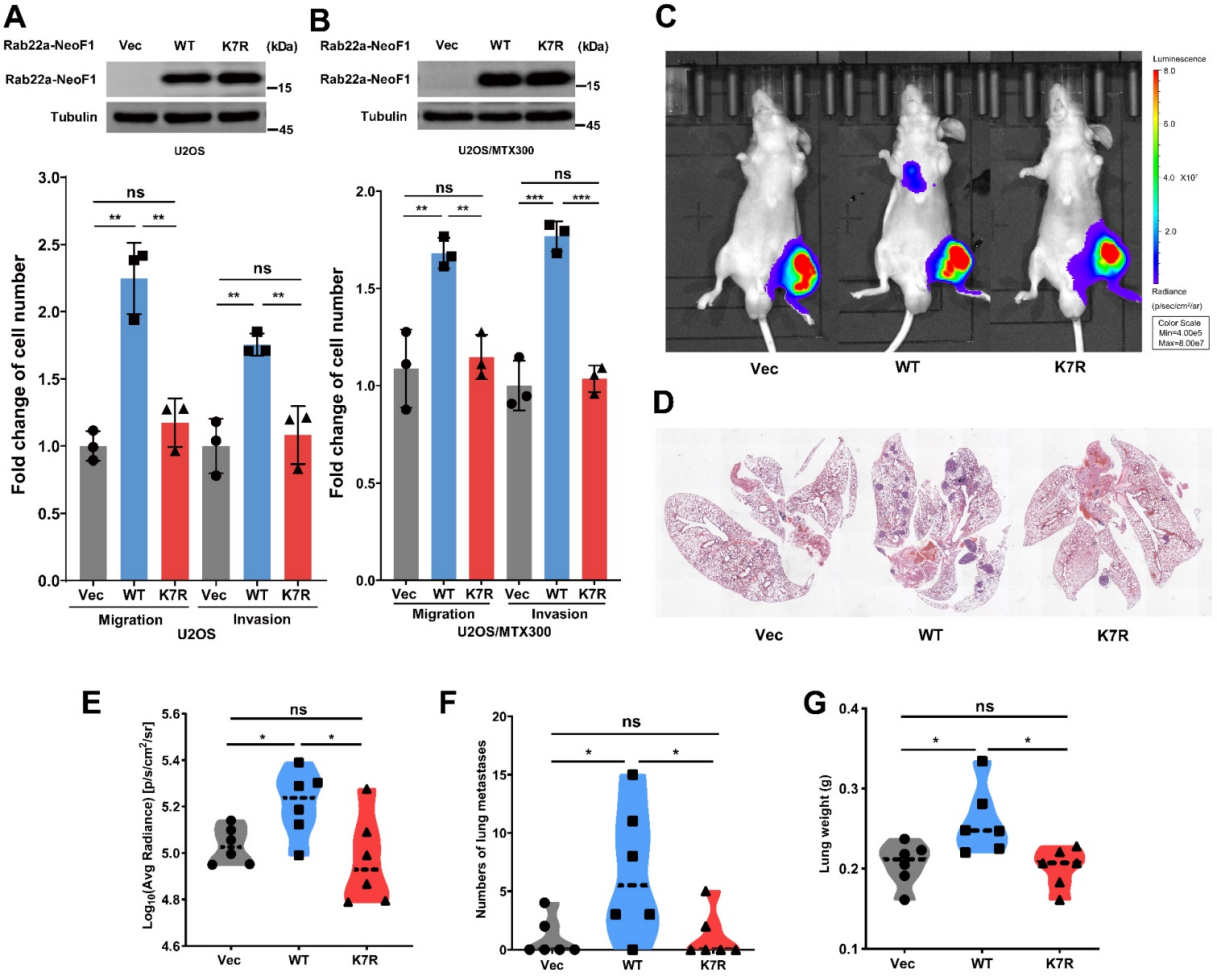
** The K7R mutant of Rab22a-NeoF1 lacks its capacities of enhancing migration, invasion and metastasis *in vitro* and *in vivo*.** (**A, B**) Quantification analyses of migration and invasion assays (lower panel) using U2OS cells or U2OS/MTX300 cells stably expressing Vector, Rab22a-NeoF1 (WT) or its K7R mutant (K7R), as indicated. The expression level of WT and K7R Rab22a-NeoF1 in U2OS cells and U2OS/MTX300 cells were shown in the upper panel*.* (**C-F**) The orthotopic osteosarcoma metastasis model *in vivo* using the U2OS/MTX300-Luc cells stably expressing Vector, Rab22a-NeoF1 (WT) or its K7R mutant (K7R), as indicated. Representative images of mice (**C**). H&E staining of the lungs from representative tumor-bearing nude mice (**D**). Quantification analyses of Log_10_(Average Radiance) of lung metastasis. (**E**) Quantification analyses of lung nodules (**F**). Quantification analyses of wet lung weight (**G**) from the nude mice used in **C.**

To investigate whether the K7 acetylation of Rab22a-NeoF1 regulates its promoting osteosarcoma metastasis, we generated their stable U2OS cell lines and U2OS/MTX300 cell lines (U2OS/MTX300 cell line is a methotrexate-resistant derivative of the U2OS human osteosarcoma cell line). Rab22a-NeoF1, but not Rab22a-NeoF1-K7R, dramatically promotes migration and invasion compared to those stably expressing Vector (**Figure 2A-B**). Next, the ectopic Rab22a-NeoF1-K7R was stably transfected into U2OS/MTX300-Luc cells, U2OS/MTX300 cells overexpressing Luciferase. Using the orthotopic osteosarcoma metastasis model *in vivo*, lung metastases of U2OS/MTX300-Luc cells stably expressing Rab22a-NeoF1, but not Rab22a-NeoF1-K7R, were dramatically enhanced compared to those stably expressing Vector (**Figure 2C-G**). These results demonstrate that the K7R mutant of Rab22a-NeoF1 lacks its capacities of enhancing migration, invasion and metastasis *in vitro* and *in vivo*, indicating that the K7 acetylation of Rab22a-NeoF1 may be crucial for its promoting functions.

### p300/CBP acetyltransferases are responsible for the K7 acetylation of Rab22a-NeoF1

**Figure 3:**
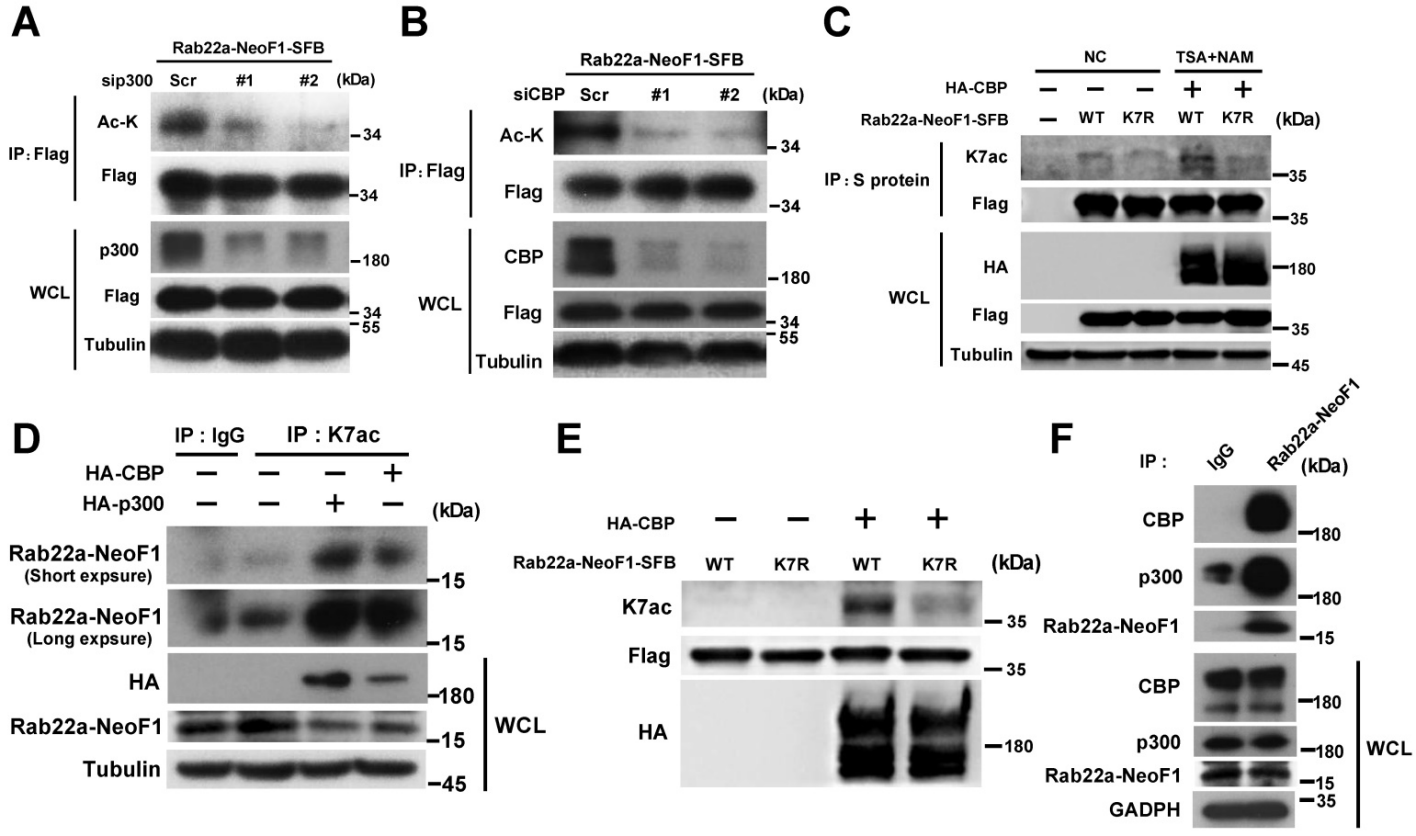
** p300/CBP acetyltransferase are responsible for the K7 acetylation of Rab22a-NeoF1.** (**A**,**B**) HEK293T cells were co-transfected Rab22a-NeoF1-SFB with the p300 (**A**) or CBP (**B**) specific siRNAs indicated acetyltransferases plasmids for 48 h, cell lysates were subjected to IP using anti-Flag agarose, and then were analyzed by Western blotting.** (C)** HEK293T cells transiently transfected Rab22a-NeoF1-SFB (WT) or its K7R mutant (K7R) with HA-CBP for 24 h were treated with both TSA (5 μM) and NAM (5 mM) for 8 h. Cell lysates were subjected to IP using anti-S protein beads, and then were analyzed by Western blotting. (**D**) ZOS-M cells were transfected with HA-p300 or HA-CBP, cell lysates were subjected to IP using anti-K7ac-Rab22a-NeoF1 antibody, and then were analyzed by Western blotting by mAb RAD5-8. (**E**) Rab22a-NeoF1-WT-SFB or the Rab22a-NeoF1-K7R-SFB mutant was incubated with HA-CBP purified from HEK293T cells in acetylation buffer for 1 h. in vitro and then analyzed using anti-K7ac-Rab22a-NeoF1 antibody by Western blotting. (**F**) ZOS-M cells were lysed and subjected to IP using mAb RAD5-8, and then were analyzed by Western blotting using anti-p300, anti-CBP or hRAD5-8-v1-R5 antibody.

To identify the acetyltransferase responsible for the K7 acetylation of Rab22a-NeoF1, HEK293T cells were co-transfected Rab22a-NeoF1 with each one of five common protein acetyltransferases, HA-p300, HA-CBP, HA-GCN5, HA-Tip60 and HA-PCAF. We found that p300/CBP significantly increased the acetylation level of Rab22a-NeoF1 compare to other acetyltransferases using anti-ac-K antibody (**Figure S2A**)**.** Consistently, the acetylation level of Rab22a-NeoF1 was significantly decreased by siRNAs targeting p300 or CBP (**Figure 3A-B**), whereas CBP significantly increased the acetylation level of Rab22a-NeoF1, but not Rab22a-NeoF1-K7R, under treatment of TSA plus NAM (**Figure 3C**)**.** More importantly, using the K7ac antibody, the K7 acetylation of endogenous Rab22a-NeoF1 was also dramatically increased in ZOS-M cells transiently transfected with CBP or p300 (**Figure 3D**), which was further supported by the in vitro acetylation of Rab22a-NeoF1 by CBP (**Figure 3E**). In addition, the association of Rab22a-NeoF1 with p300 or CBP was detected at their ectopic and endogenous levels (**Figure 3F, S2B-C**), and such interactions were not altered by the treatments of their inhibitors, such as salicylate and C646, in ZOS-M cells (**Figure S2D-E**). Collectively, these results reveal that the K7 of Rab22a-NeoF1 is acetylated by p300/CBP.

### C646 inhibits migration, invasion and lung metastases induced by Rab22a-NeoF1 *in vitro* and *in vivo*

**Figure 4:**
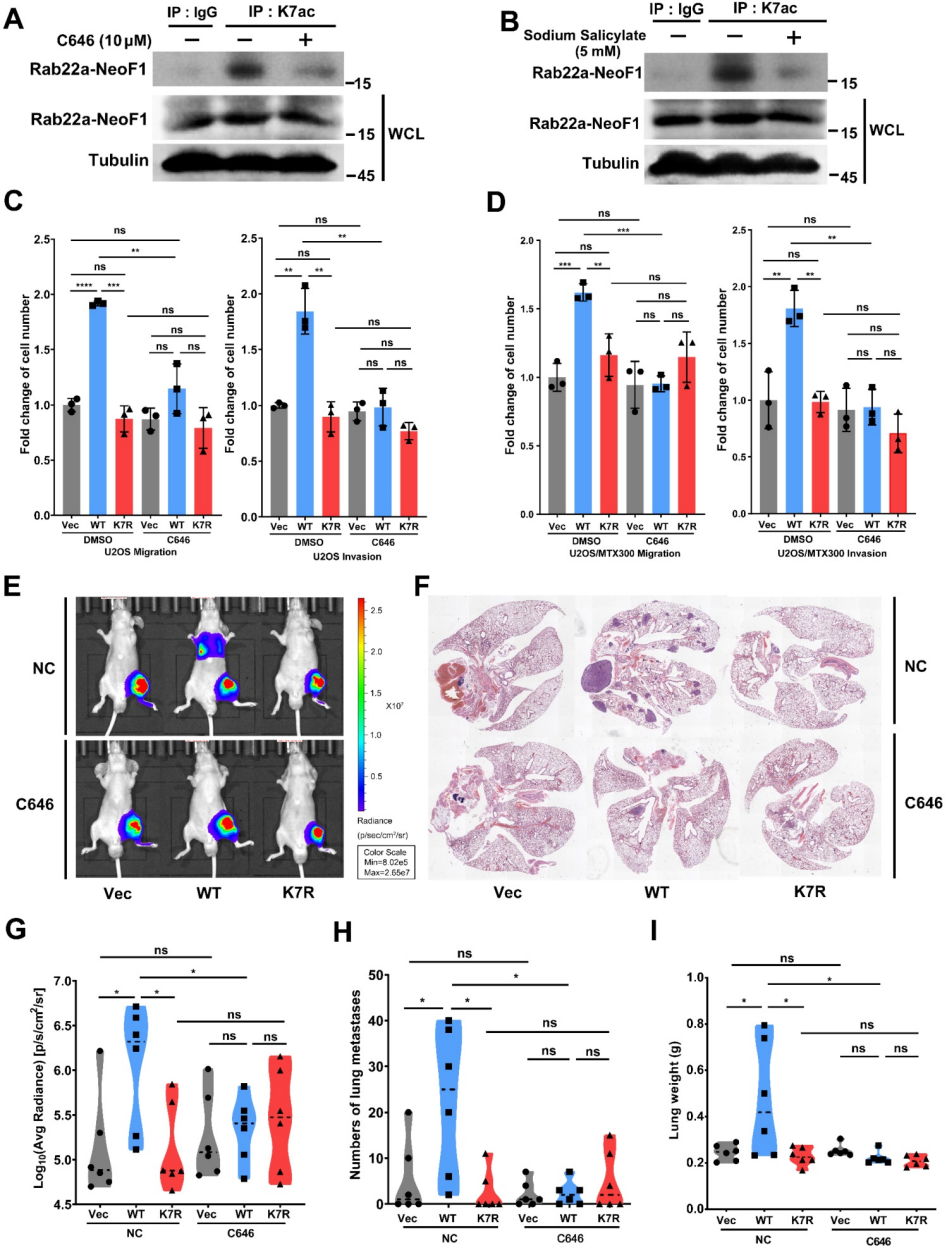
** C646 inhibits migration, invasion and the lung metastases induced by Rab22a-NeoF1 *in vitro* and *in vivo*.** (**A, B**) ZOS-M cells were treated with 5 µM C646 (**A**) or 5 mM salicylate (**B**) for 24 h, cell lysates were subjected to IP using anti-K7ac-Rab22a-NeoF1 antibody, and then were analyzed by Western blotting by mAb RAD5-8. (**C, D**) Quantification analyses of migration and invasion assays using U2OS cells (**C**) or U2OS/MTX300 (**D**) cells stably expressing Vector, Rab22a-NeoF1 (WT) or its K7R mutant (K7R), as indicated, with or without the treatment of 5 µM C646 for 24 h. (**E-H**) The orthotopic osteosarcoma metastasis model *in vivo* using the U2OS/MTX300-Luc cells stably expressing Vector, Rab22a-NeoF1 (WT) or its K7R mutant (K7R), as indicated. After cell injection into mice for 3 weeks, the mice were intraperitoneally injected with C646 (10 mg/kg/d) or the control (7.7% DMSO+40% PEG300+ddH2O) daily for 14 days. Lung metastasis were detected by in vivo fluorescent imaging two months after cell injection. Representative images of mice (**E**). H&E staining of the lungs from representative tumor-bearing nude mice (**F**). Quantification analyses of Log_10_(Average Radiance) of lung metastasis (**G**). Quantification analyses of lung nodules (**H**). Quantification analyses of wet lung weight (**I**) from the nude mice used in **E.**

Both C646 and sodium salicylate (SA) are specific small molecular inhibitors of p300/CBP acetyltransferases by directly binding to p300/CBP to compete with acetyl-CoA 24, 25. C646 or SA suppressed the acetylation level of ectopic Rab22a-NeoF1, but not Rab22a-NeoF1-K7R, in cells (**Figure S3A-B**). Moreover, C646 or SA reduced the K7 acetylation level of endogenous Rab22a-NeoF1 in ZOS-M cells (**Figure 4A-B**). Since SA, but not C646, inhibited cell viability of osteosarcoma cells (**Figure S3C-D**), we tried to explore whether C646 could repress the promoting functions of Rab22a-NeoF1. As shown in **Figure 4C-D**, C646 impaired migration and invasion of both U2OS cells and U2OS/MTX300 cells stably expressing Rab22a-NeoF1, but not either Rab22a-NeoF1-K7R or Vector. Moreover, using the orthotopic osteosarcoma metastasis model *in vivo*, intraperitoneal administration of C646 into mice diminished lung metastases of U2OS/MTX300-Luc stably expressing Rab22a-NeoF1, but neither Rab22a-NeoF1-K7R nor Vector (**Figure 4E-I**). These results illustrate that pharmaceutically targeting the K7 acetylation of Rab22a-NeoF1 (e. g., C646) may be efficient to prevent osteosarcoma lung metastasis induced by Rab22a-NeoF1.

### Rab22a-NeoF1 K7 deacetylation is synergistically regulated by HDAC6 and SIRT1 in cells

**Figure 5:**
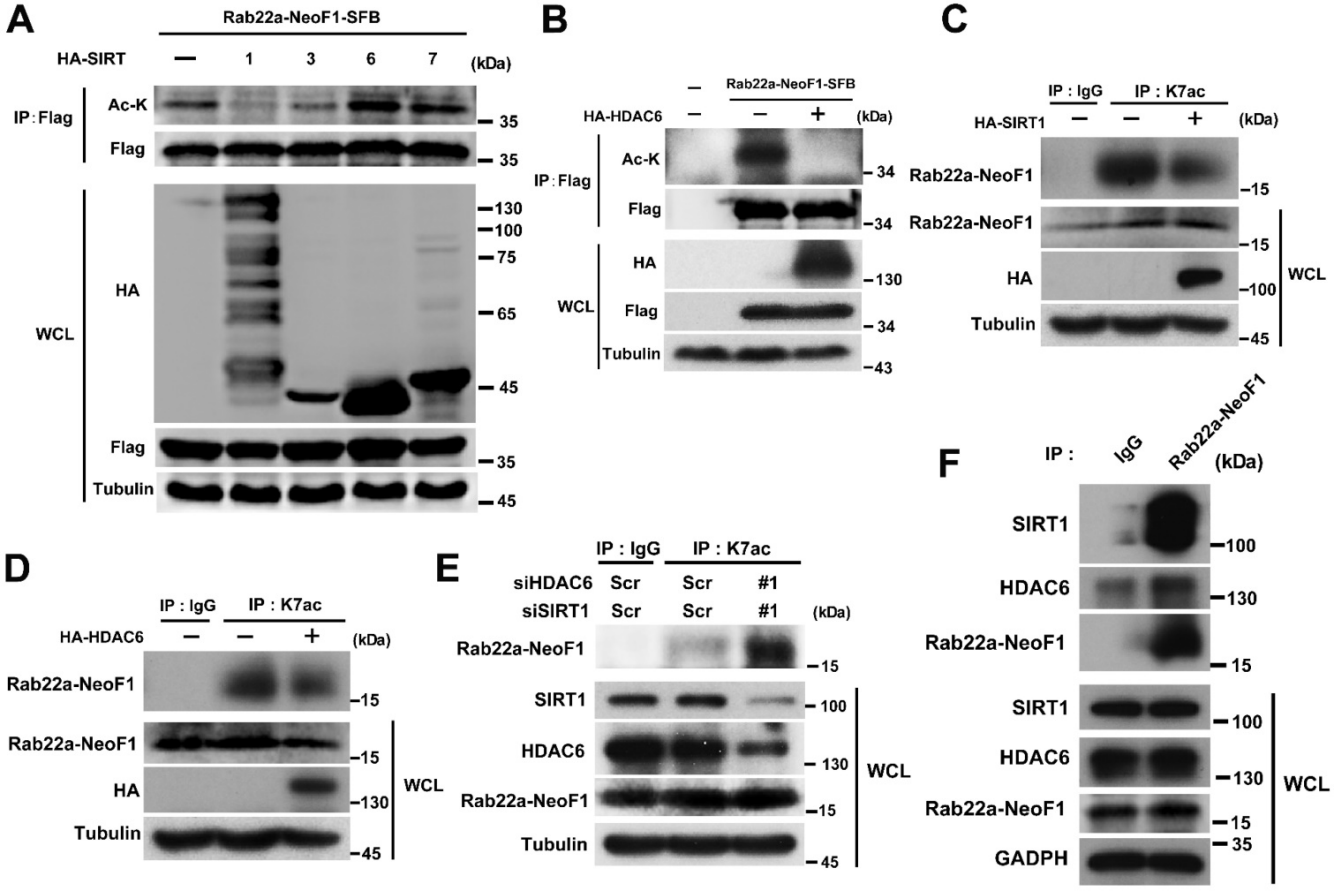
** Rab22a-NeoF1 K7 deacetylation is regulated by HDAC6 and SIRT1.** (**A, B**) HEK293T cells were co-transfected Rab22a-NeoF1-SFB with the indicated HA-tagged histone deacetylases plasmids for 48 h, cell lysates were subjected to IP using anti-Flag agarose, and were then analyzed by Western blotting. (**C, D**) ZOS-M cells were transfected with HA-SIRT1 (**C**) or HA-HDAC6 (**D**) for 48 h, cell lysates were subjected to IP using anti-K7ac-Rab22a-NeoF1 antibody, and then were analyzed by Western blotting by mAb RAD5-8. (**E**) ZOS cells were transfected with siRNA targeting SIRT1 and HDAC6 for 48 h, cell lysates were subjected to IP using anti-K7ac-Rab22a-NeoF1 antibody, and then were analyzed by Western blotting by mAb RAD5-8. (**F**) ZOS-M cells were lysed and subjected to IP using mAb RAD5-8, and then were analyzed by Western blotting using anti-SIRT1, anti-HDAC6 or hRAD5-8-v1-R5 antibody.

Next, we tried to look into which deacetylase is involved in the K7 acetylation of Rab22a-NeoF1. Since both the sirtuins inhibitor NAM and HDAC inhibitor TSA enhanced the acetylation level of Rab22a-NeoF1 (**Figure 1B-D**). HEK293T cells were co-transfected Rab22a-NeoF1 with each HA-tagged sirtuin (SIRT1-7), and we found that Rab22a-NeoF1 could form a complex with SIRT1, SIRT3, SIRT6 or SIRT7, but not with SIRT2, SIRT4 or SIRT5 (**Figure S4A**). However, SIRT1 and SIRT3, but not SIRT6 or SIRT7, were able to decrease the acetylation level of Rab22a-NeoF1 (**Figure 5A**). Given that SIRT3 mainly localizes in the mitochondria 26, 27, while SIRT1 has a localization of cytoplasm and nuclear 28, 29, and Rab22a-NeoF1 is mainly located in cytoplasm. Therefore, we speculate that SIRT1 may be the main sirtuin to deacetylate the K7 acetylation of Rab22a-NeoF1. Using the same strategy, we found that only HDAC6 among the eleven HDAC members was immunoprecipitated with Rab22a-NeoF1 (**Figure S4B**), and that overexpression of HDAC6 could diminish the acetylation level of Rab22a-NeoF1 (**Figure 5B**). More importantly, overexpression of SIRT1 or HDAC6 impeded the K7ac level of endogenous Rab22a-NeoF1 in ZOS-M cells (**Figure 5C-D**). Knock down of SIRT1 and HDAC6 using siRNA up-regulated the level of endogenous K7ac-Rab22a-NeoF1 in ZOS cells (**Figure 5E**). Supportively, the association of Rab22a-NeoF1 with SIRT1 or HDAC6 was detected at their ectopic and endogenous levels (**Figure 5F, S4C-D**), and such interactions were not altered by the treatments of TSA plus NAM in ZOS-M cells (**Figure S4E**). Taken together, we conclude that SIRT1 and HDAC6 are responsible for the deacetylation of Rab22a-NeoF1 K7.

### The inhibition of p300/CBP or the K7R mutant impairs the interaction of Rab22a-NeoF1 with SmgGDS607 to inhibit its activating RhoA

**Figure 6:**
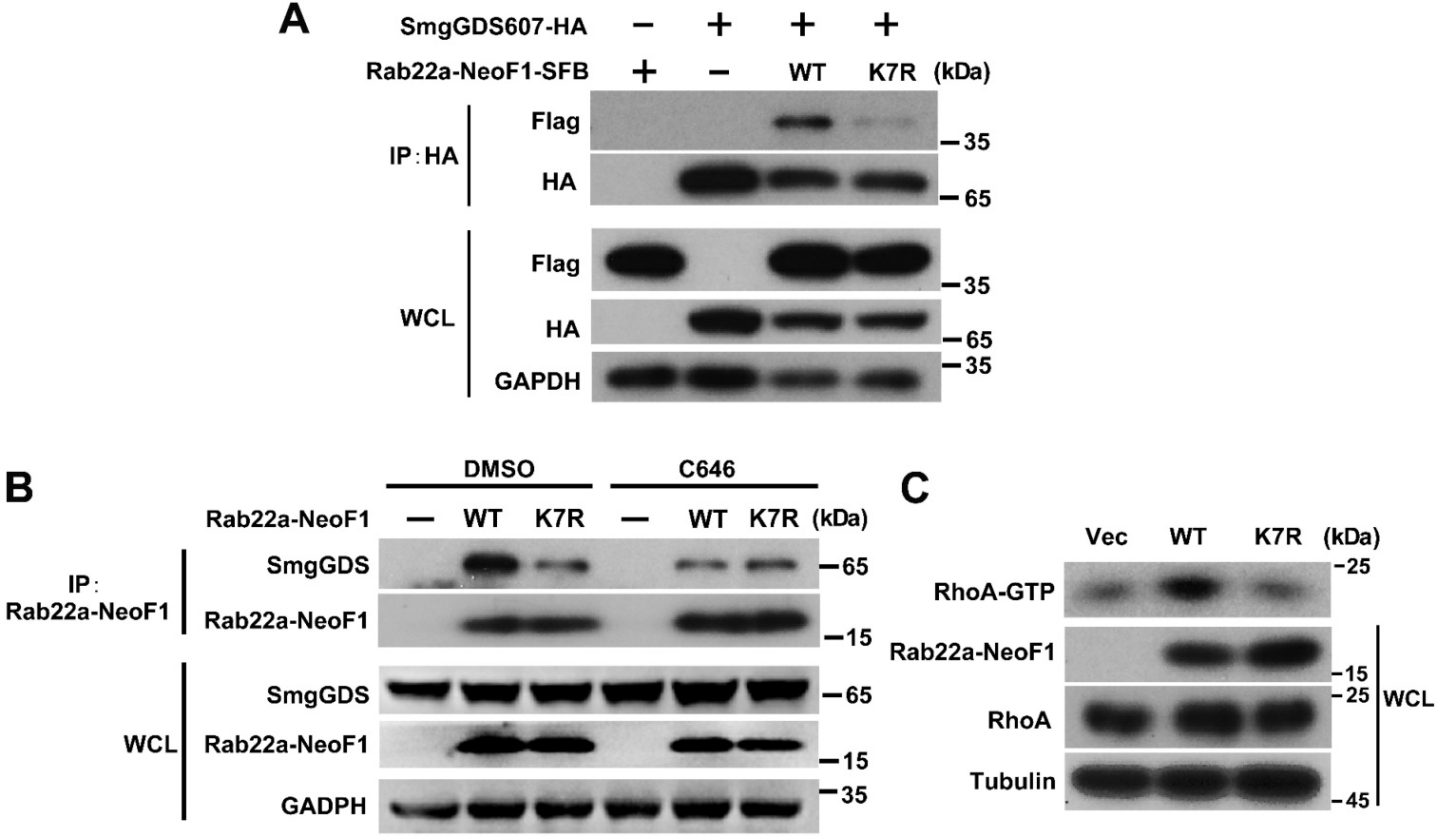
**The inhibition of p300/CBP or the K7R mutant impairs the interaction of Rab22a-NeoF1 with SmgGDS607 and the activation of RhoA by Rab22a-NeoF1.** (**A**) HEK293T cells were co-transfected SmgGDS607-HA with Rab22a-NeoF1-SFB (WT) or its K7R mutant (K7R), as indicated. Cell lysates were subjected to IP using anti-HA, and were then analyzed by Western blotting. (**B**) U2OS cells stably overexpressing Rab22a-NeoF1-SFB (WT) or its K7R mutant (K7R) were treated with 10 μM C464 for 24 h, as indicated, cell lysates were subjected to IP using mAb RAD5-8 and/or Western blotting using hRAD5-8-v1-R5 antibody. (**C**) U2OS/MTX300 cells stably overexpressing Vector, Rab22a-NeoF1-SFB (WT) or its K7R mutant (K7R) were subjected to the RhoA GTPase activation assay.

Finally, we sought to explore how the K7 acetylation affects the functions of Rab22a-NeoF1. Given that Rab22a-NeoF1 fusion protein activates RhoA through constitutively binding to SmgGDS607 utilizing R4 and K7 8, we surmised that the K7 acetylation of Rab22a-NeoF1 may affect its binding to SmgGDS607. It was the case that both binding to SmgGDS607 and the RhoA-GTP level were dramatically decreased in cells stably expressing Rab22a-NeoF1-K7R compared to those stably expressing Rab22a-NeoF1 **(Figure 6A-C, S5A-B)**. Consistently, C646 or SA treatment could also suppress the association of SmgGDS607 with Rab22a-NeoF1, but not with the Rab22a-NeoF1-K7R mutant (**Figure 6B, S5B**). These results illustrate that the K7 acetylation plays a critical role in its interaction with SmgGDS607 and in its subsequent activation of RhoA to promote osteosarcoma lung metastasis.

## Discussion

In this report, we found that the K7 of Rab22a-NeoF1, acetylated by p300/CBP while de-acetylated by both HDAC6 and SIRT1, plays a key role in its binding to SmgGDS607, and consequently affects its activation of RhoA and promotion of cell migration, invasion and lung metastasis in osteosarcoma. This regulation may provide a strategy to treat osteosarcoma patients with metastasis by targeting this acetylation event (e.g., C646 and SA).

**Figure 7:**
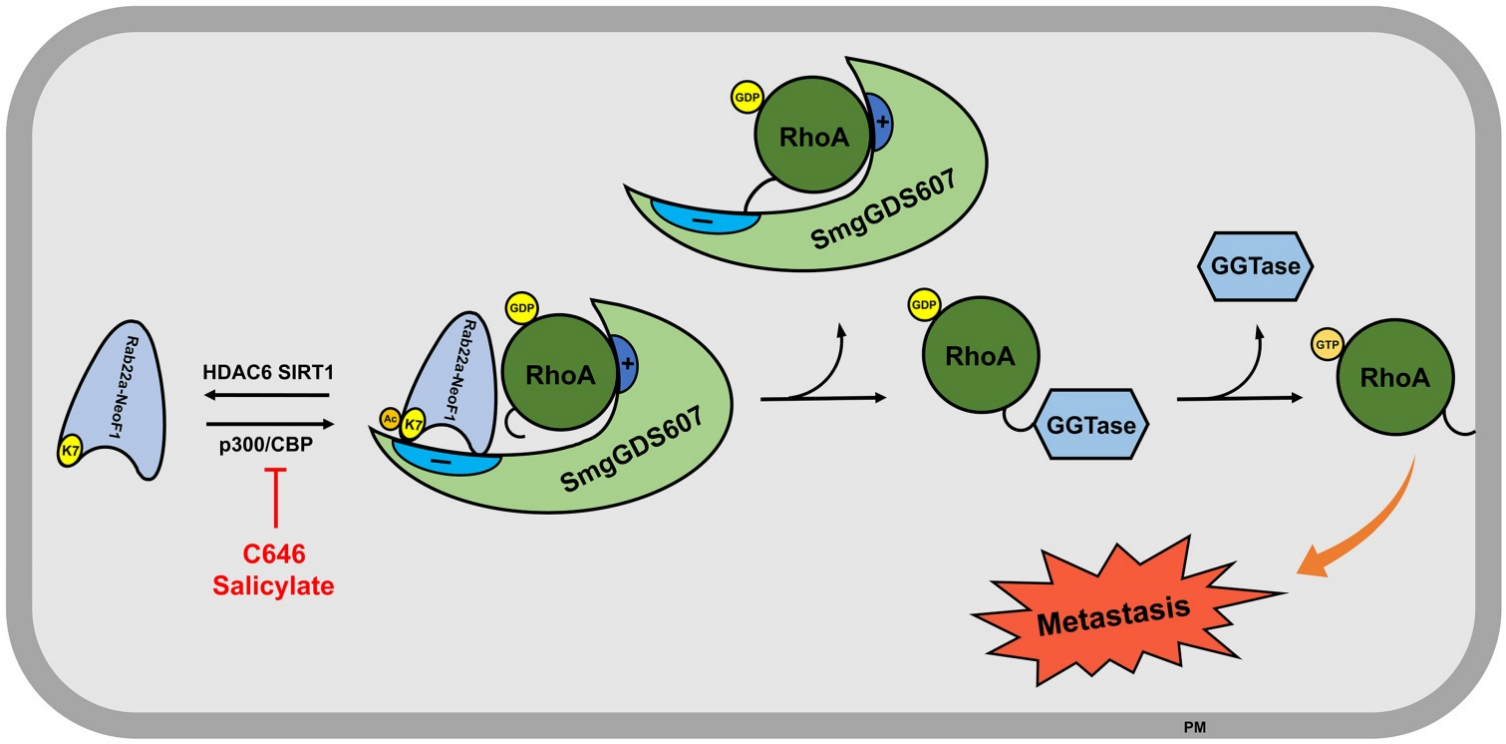
** The proposed model for the K7 acetylation of Rab22a-NeoF1.** Acetylation of Rab22a-NeoF1 at K7, which is acetylated by p300/CBP and deacetylated by both SIRT1 and HDAC6, may be required for its association with SmgGDS607, resulting in more RhoA-GTPase to promote lung metastasis. C646 or salicylate targeting p300/CBP may be benefit for the osteosarcoma patients who are positive for Rab22a-NeoF1.

The functional fusions of RAB22A^1-38^ with different genes have been also found in multiple cancer cell lines and tissues, such as breast cancer cell line BT-474 and tumor tissues, as well as HCC2935 lung cancer line 8. Given that the RAB22A^1-38^ plays a decisive role in promoting metastasis for this type of fusion proteins, as the formation of such fusion proteins (e.g., Rab22a-NeoF1) results in the surface-exposure of K7, which is blocked by the main chain atoms of Met73, Try74 and Arg76 in RAB22A. As illustrated in **Figure 7**, Rab22a-NeoF1 is acetylated at K7; such an acetylation facilitates its interaction with SmgGDS607 and leads to the excessive activation of RhoA GTPase to be transferred on membrane, which in turn promotes metastasis in osteosarcoma. This acetylation of K7 may be extended for all fusion proteins containing RAB22A^1-38^ in cancers, and may affect their functions, such as binding to SmgGDS607, activation of RhoA, promotion of migration, invasion and metastasis. Notably, the K7 acetylation of Rab22a-NeoF1 may not affect its stability, as the ubiquitination levels of both Rab22a-NeoF1 and its K7R mutant are similar, and are not affected by TSA plus NAM treatment (**Figure S1F**).

Numerous studies had identified that deregulated acetylation is associated with various human diseases including developmental disorders, inflammation, immunity, neurological disorders, metabolic diseases and cancers. Thus, protein acetylation had been considered as attractive therapeutic targets, and small molecular inhibitors of KDACs, KATs and bromodomain proteins (acetyl-lysine readers) have emerged as attractive therapeutic candidates 30, 31, and some of which have been assessed in the clinic as therapies for diseases like advanced leukaemia, myelodysplastic syndromes, neurological diseases and immune disorders. HDACis like vorinostat, belinostat, panobinostat, romidepsin, valproic acid and sodium butyrate have been approved by FDA for the treatment of cutaneous T cell lymphoma, peripheral T cell lymphoma, multiple myeloma or neurological disorders 30, 32, 33. SA and C646 are both specific small molecular inhibitors of p300/CBP acting by inhibiting the acetyltransferase activity through binding with p300/CBP competing with acetyl-CoA directly 24, 25. As reported by Yujie Liu. et al, conditional deletion or C646 pharmacologic inhibition of p300 in Foxp3+ Treg cells increased T cell receptor-induced apoptosis in Treg cells, leading to limited AE17 tumor growth in immunocompetent mice, indicating that C646 is a promising therapeutic candidate for cancer immunotherapy 34. Sang-Won Min. et al had identified that p300-induced tau K174 acetylation, which is an early change in AD brains, can be inhibited by C646 and SA; Administration of SA rescued tau-induced memory deficits and prevented hippocampal atrophy in mouse models, suggesting that Targeting tau acetylation could be a new therapeutic strategy against human tauopathies 16. In this study, we provide evidence that C646 antagonizes the enhancement of migration, invasion and lung metastasis induced by Rab22a-NeoF1 in osteosarcoma cells and in the orthotopic osteosarcoma metastasis model *in vivo*. We propose that targeting acetylation using C646 or SA may benefit cancer patients, in particular osteosarcoma patients, who are positive for the RAB22A^1-38^.

## Supplementary Material

Supplementary figures and tables.

## Figures and Tables

**Table 1:** peptides

No	PTM Type	PTM site	Sequence	Note
1	Acetyl	Lys7	ALREL-(acetyl)K-VSLLGDC	Immunization
2			ALREL-(acetyl)K-VSLLGDTGVC	Immunization
3			ALRELKVSLLGDC	Depletion

## Abbreviations

K7: lysine 7
MS: mass spectrometry
TSA: Trichostatin A
NAM: Nicotinamide (Vitamin B3)
Ac-K: lysine acetylation
K7ac: lysine 7 acetylation
Mono-me-K: lysine mono-methylation
Di-me-K: lysine di-methylation
Tri-me-K: lysine tri-methylation
SA: sodium salicylate
Luc: luciferase
