# Supplementary material for: Acetylation dependent functions of Rab22a-NeoF1 Fusion Protein in Osteosarcoma
Source: Theranostics. 2020 Jun 19;10(17):7747–57. doi: 10.7150/thno.46082 (PMC7359080; doi:10.7150/thno.46082)
Supplement: Supplementary file 1 — Supplementary figures and tables. [file thnov10p7747s1.pdf]

Supplementary data

Figure S1.

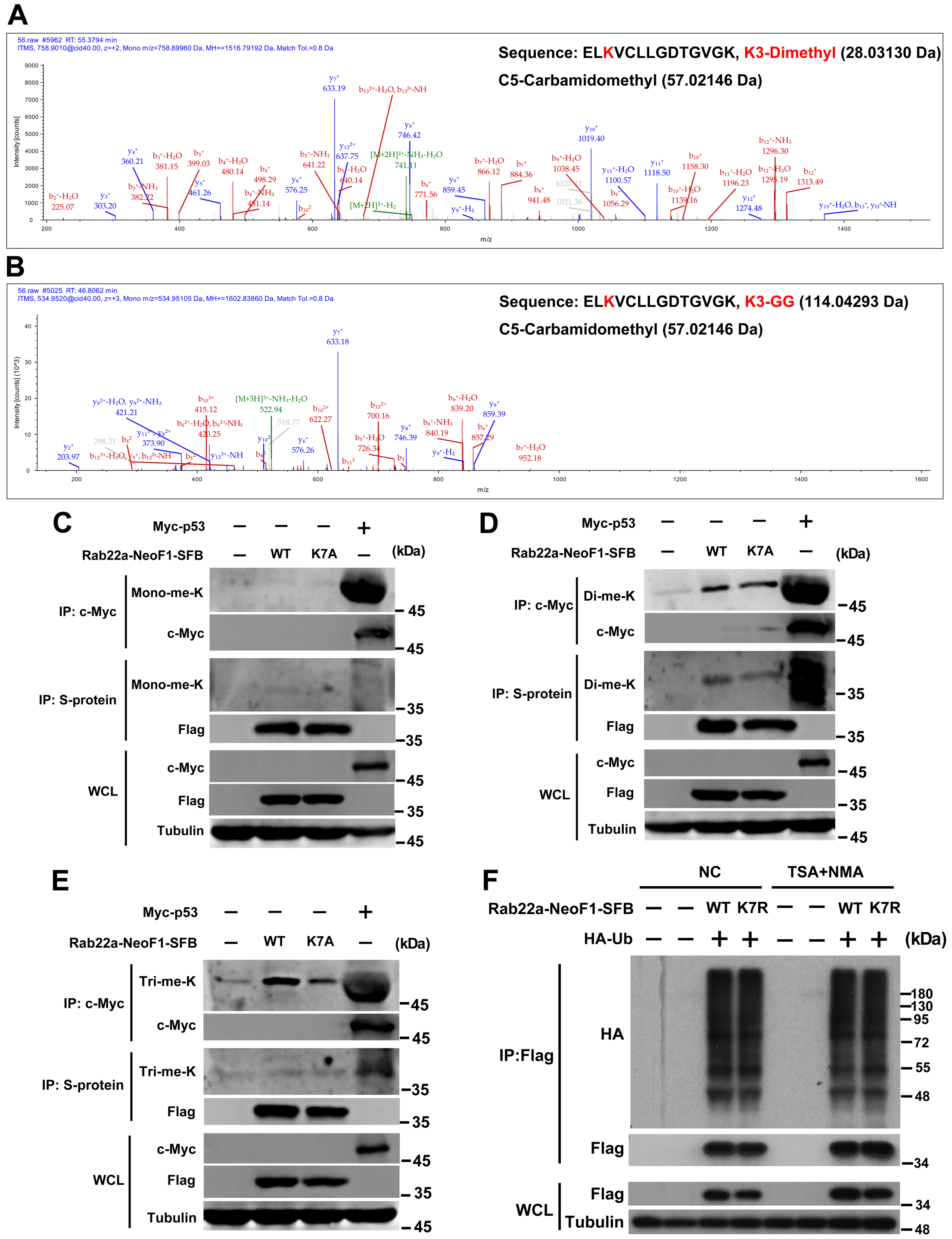

Figure S1. Identification of possible post-translational modification of K7 within Rab22a-NeoF1.

(**A, B**) HEK293T cells transfected with SFB-tagged Rab22a-NeoF1 for 48 h, and cell lysates were subjected to immunoprecipitation (IP) using anti-Flag agarose, the IP complex was then analyzed by mass spectrometry. MS spectra of K-GG (**A**), K-dimethyl (**B**) containing the 5-ELKVCLLGDTGVGK-18 peptide obtained after trypsin digestion of the IP complex. (**C-E**) HEK293T cells were transiently transfected with Rab22a-NeoF1-SFB (WT) or its K7A mutant (K7A), as well as Myc-p53 that was used as the positive control for methylation. After 48 h, cell lysates were subjected to IP using anti-S protein beads or anti-c-Myc agarose, and then were analyzed by Western blotting using mono-methyl (**C**), di-methyl (**D**) or tri-methyl (**E**) lysine antibody. (**F**) HEK293T cells transiently transfected Rab22a-NeoF1-SFB (WT) or its K7R mutant (K7R) with HA-Ub for 24 h were treated with both TSA (5 μM) and NAM (5 mM) for 8 h. Cell lysates were subjected to IP using anti-Flag agarose, and then were analyzed by Western blotting.

Figure S2

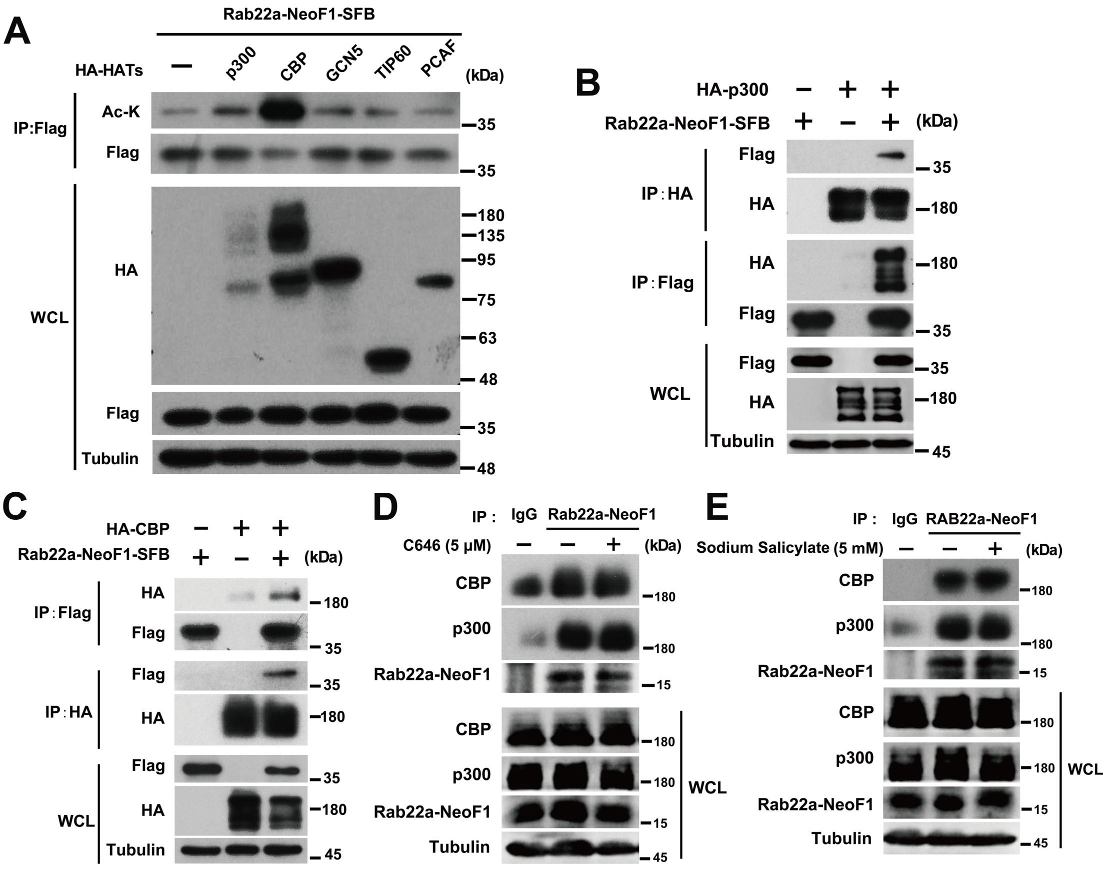

Figure S2. p300/CBP significantly increased the acetylation level of Rab22a-NeoF1, and interacts with Rab22a-NeoF1 at their ectopic and endogenous levels in cells

(**A**)HEK293T cells were co-transfected Rab22a-NeoF1-SFB with the indicated acetyltransferases plasmids for 48 h, cell lysates were subjected to IP using anti-Flag agarose, and then were analyzed by Western blotting. (**B,C**) HEK293T cells were transiently transfected Rab22a-NeoF1-SFB with HA-p300 (**B**), HA-CBP (**C**), for 48 h. Cell lysates were subjected to IP using anti-HA , anti-Flag agarose, and then were analyzed by Western blotting. (**D,E**) ZOS-M cells were treated with C646 (5 μM) or sodium salicylate (5 mM) for 24 h. Cell lysates subjected to IP using mAb RAD5-8 and then were analyzed by Western blotting using anti-p300,CBP antibody or hRAD5-8-v1-R5 antibody.

Figure S3

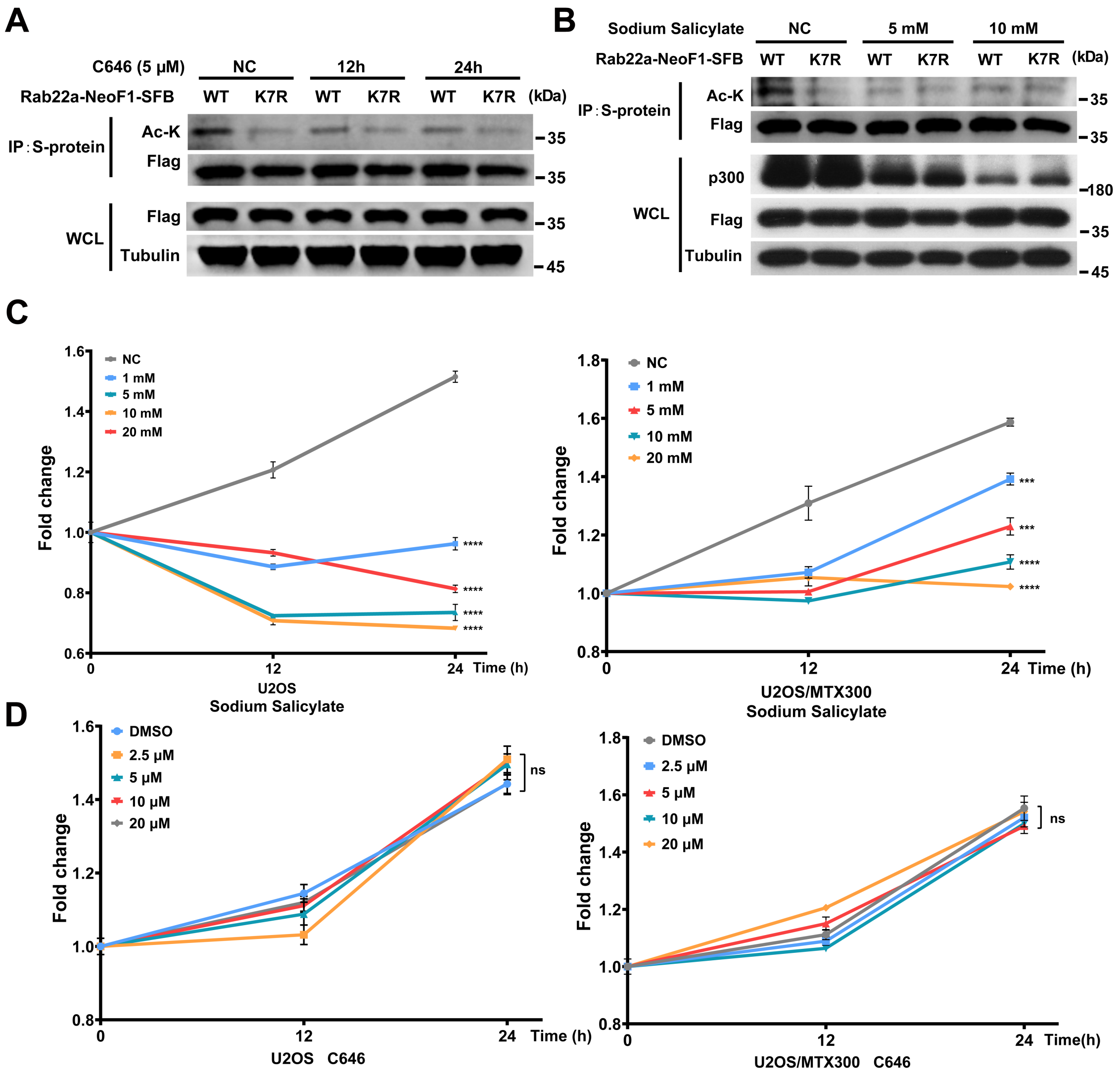

Figure S3. Effects of C646 or SA on both acetylation of Rab22a-NeoF1 and cell viability in osteosarcoma cells

(**A, B**) HEK293T cells transiently transfected with Rab22a-NeoF1-SFB or its K7R mutant for 24 h were treated with the indicated concentrations of C646 and sodium salicylate for the time points (**A**) and 24 h (**B**), as indicated, respectively. Cell lysates were subjected to IP using anti-S protein beads, and were then analyzed by Western blotting. (**C, D**) U2OS (left) and U2OS/MTX300 (right) cells were treated with different doses of C646 (**C**) or SA (**D**) for 12 or 24 h, cell viability was then measured by MTT assay.

Figure S4.

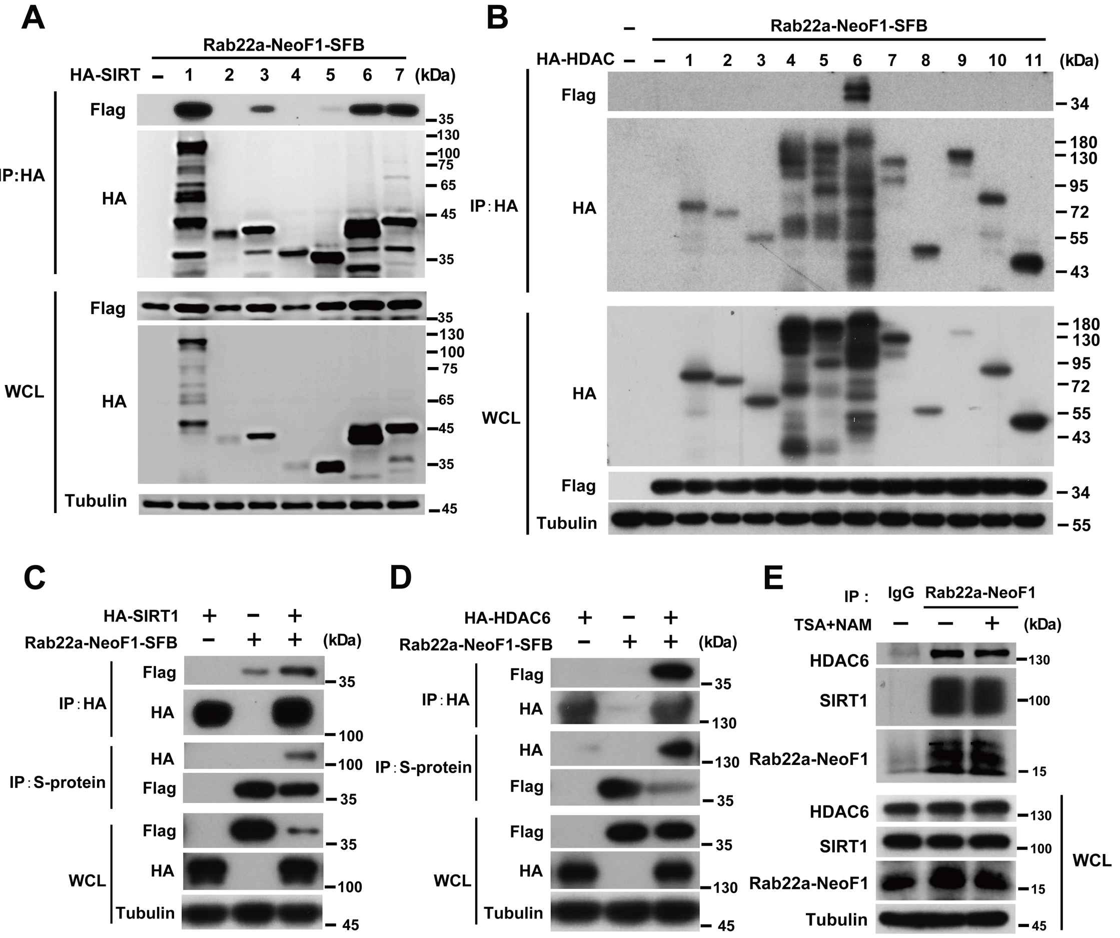

Figure S4. Both HDAC6 and SIRT1 interact with Rab22a-NeoF1 at their ectopic and endogenous levels in cells

(**A, B**) HEK293T cells were co-transfected Rab22a-NeoF1-SFB with the indicated HA-tagged sirtuins deacetylase plasmids for 48 h, cell lysates were subjected to IP using or anti-HA agarose, and were then analyzed by Western blotting. (**C, D**) HEK293T cells were transiently transfected Rab22a-NeoF1-SFB with HA-SIRT1 (**C**) or HA-HDAC6 (**D**) for 48 h. Cell lysates were subjected to IP using anti-HA or anti-S protein beads, and then were analyzed by Western blotting. (**E**) ZOS-M cells were treated with both TSA (5 μM) and NAM (5 mM) for 8 h. Cell lysates were subjected to IP using mAb RAD5-8, and then were analyzed by Western blotting using anti-HDAC6, anti-SIRT1 or hRAD5-8-v1-R5 antibody.

Figure S5.

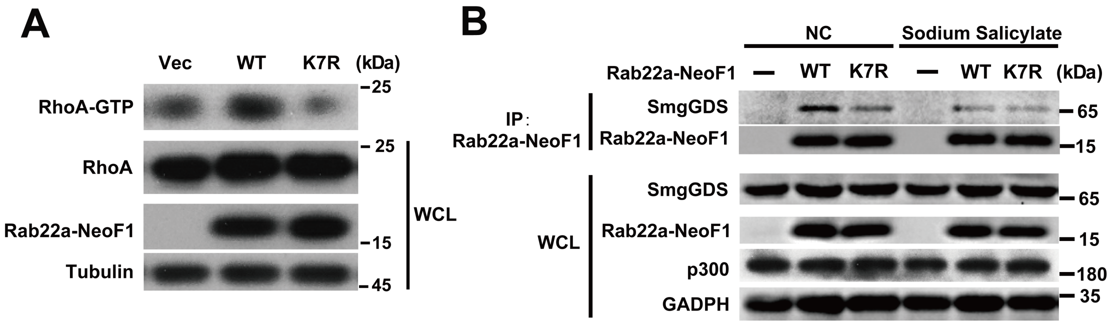

Figure S5. The K7R mutant of Rab22a-NeoF1 impairs its binding to SmgGDS-607 and reduces its Rho-GTP level.

(**A**) U2OS cells stably overexpressing Vector, Rab22a-NeoF1-SFB (WT) or its K7R mutant (K7R) were subjected to the RhoA GTPase activation assay**.** (**B**) U2OS cells stably overexpressing Vector, Rab22a-NeoF1-SFB (WT) or its K7R mutant (K7R) were treated with or without 5 mM salicylate for 24 h, as indicated, cell lysates were subjected to IP using mAb RAD5-8 and/or Western blotting using hRAD5-8-v1-R5 antibody.

The oligo nucleotide sequences of the primers for cloning are as follows: (16 bases of sequence homology are shown in capital letters)

p3.1-HA-HDAC1	F:ATTACGCTTCGGATCCgcgcagacgcagggcacccg
	R:GCCCTCTAGACTCGAGtcaggccaacttgacctcct
p3.1-HA-HDAC2	F:ATTACGCTTCGGATCCgcgtacagtcaaggaggcgg
	R:GCCCTCTAGACTCGAGtcaggggttgctgagctgtt
p3.1-HA-HDAC3	F:ATTACGCTTCGGATCCgccaagaccgtggcctatttc
	R:GCCCTCTAGACTCGAGttaaatctccacatcgctttc
p3.1-HA-HDAC4	F:ATTACGCTTCGGATCCagctcccaaagccatccaga
	R:GCCCTCTAGACTCGAGctacaggggcggctcctctt
p3.1-HA-HDAC5	F:ATTACGCTTCGGATCCaactctcccaacgagtcggc
	R:GCCCTCTAGACTCGAGtcacagggcaggctcctgct
p3.1-HA-HDAC6	F:ATTACGCTTCGGATCCacctcaaccggccaggattc
	R:GCCCTCTAGACTCGAGttagtgtgggtggggcatat
p3.1-HA-HDAC7	F:ATTACGCTTCGGATCCcacagccccggcgctgatgg
	R:GCCCTCTAGACTCGAGttagagattcataggttctt
p3.1-HA-HDAC8	F:ATTACGCTTCGGATCCgaggagccggaggaaccggc
	R:GCCCTCTAGACTCGAGctagaccacatgcttcagat
p3.1-HA-HDAC9	F:ATTACGCTTCGGATCCcacagtatgatcagctcagt
	R:GCCCTCTAGACTCGAGttaagagaactttaaagaca
p3.1-HA-HDAC10	F:ATTACGCTTCGGATCCgggaccgcgcttgtgtaccatg
	R:GCCCTCTAGACTCGAGtcaagccaccaggtgaggatgg
p3.1-HA-HDAC11	F:ATTACGCTTCGGATCCctacacacaacccagctgta
	R:GCCCTCTAGACTCGAGtcagggcactgcagggggaa
p3.1-HA-SIRT1	F:ATTACGCTTCGGATCCgcggacgaggcggccctcgcc
	R:GCCCTCTAGACTCGAGctatgatttgtttgatggat
p3.1-HA-SIRT2	F:ATTACGCTTCGGATCCgcagagccagacccctctca
	R:GCCCTCTAGACTCGAtcactggggtttctccctct
p3.1-HA-SIRT3	F:ATTACGCTTCGGATCCgcgttctggggttggcgcgc

	R:GCCCTCTAGACTCGAGctatttgtctggtccatcaa
p3.1-HA-SIRT4	F:ATTACGCTTCGGATCCaagatgagctttgcgttgactt
	R:GCCCTCTAGACTCGAGtcagcatgggtctatcaaaggc
p3.1-HA-SIRT5	F:ATTACGCTTCGGATCCcgacctctccagattgtccc
	R:GCCCTCTAGACTCGAGttaagaaacagtttcattttc
p3.1-HA-SIRT6	F:ATTACGCTTCGGATCCtcggtgaattacgcggcggggc
	R:GCCCTCTAGACTCGAGtcagctggggaccgccttgg
p3.1-HA-SIRT7	F:ATTACGCTTCGGATCCgcagccgggggtctgagccg
	R:GCCCTCTAGACTCGAGttacgtcactttcttcctttttg
Rab22a-NeoF1-K7R	F：gcgctgagggagctc**cga**gtgtgtctgctcggggatacaggtgta
	R：tacacctgtatccccgagcagacacac**tcg**gagctccctcagcgc
Rab22a-NeoF1-K7A	F：gcgctgagggagctc**gcg**gtgtgtctgctcggggatacaggtgta
	R：tacacctgtatccccgagcagacacac**cgc**gagctccctcagcgc

siRNA

All siRNA were produced by RIBOBIO. The oligo nucleotide sequences of the targeted sequences are as follows:

**Genes**	**Targeted sequences**
p300	ACTGGATTAAGTTTGATAAAT
	CAAGGTTGATTACTTATAAAT
CBP	ACTGGTTGCCTATGCTAAGAA
	CATGGACCTTTCTACCATCAA
SIRT1	GATGATCAAGAGGCAATTAAT
HDAC6	CCTCACTGATCAGGCCATATT

Methods

Transfection and lentivirus production

Transient transfection of plasmids was performed using Lipofectamine 2,000 according to the manufacturer's instructions (Invitrogen). To generate lentiviruses, HEK293T-packaging cells were transfected with empty vector (pSIN-puro), pSIN-Rab22a-NeoF1 or pSIN-Rab22a-NeoF1-K7R constructs together with PSA and PIG (pSIN constructs:PSA:PIG=3:2:1) using Lipofectamine 2,000. Media were changed 6-7 h after transfection, and the cell supernatant was collected after 48 h. The cleared supernatant was filtered through a 0.45 mm filter, aliquoted and stored at -80°C until to be used.

Generation of stable cell lines

Cells were infected by lentivirus supernatant in the presence of 8 mg/ml Polybrene for 18-24 h. 48 h after infection, the cells were selected using cell medium containing 0.5 mg/ml puromycin.

Immunoblotting and immunoprecipitation

For Western blotting, cells were lysed in RIPA lysis buffer containing protease inhibitor and phosphatase inhibitor cocktails. Lysates were cleared by centrifugation at 12,000 rpm for 20 min at 4°C. For immunoprecipitation, the lysate were first incubated with anti-Flag agarose or anti-S-protein beads overnight at 4°C, and then the precipitates were washed five times with cold RIPA buffer and were eluted with 5 X SDS-PAGE. After SDS-PAGE, the proteins were transfered from the gel to the membrane. The membrane was blocked in PBST with 5% nonfat milk for 1-4 h in room temperature. Incubate the membrane with appropriate dilutions of primary antibody in antibody dilution buffer overnight at 4°C. Wash the membrane with PBST for three times. Incubate the membrane with the recommended dilution of conjugated secondary antibody in 5% nonfat milk blocking buffer at room temperature for 1 h. Wash the membrane with PBST for three times. Acquire image using darkroom development techniques for chemiluminescence, or normal image scanning methods for colorimetric detection.

Mass spectrometric analysis

HEK293T cells transfected with SFB-tagged Rab22a-NeoF1 expression plasmids for 48 h were collected and lysed with RIPA buffer, and the cell lysates were immunoprecipitated with anti-Flag agarose beads. The Flag peptide-eluted material was resolved by 10% SDS–PAGE. The Rab22a-NeoF1 bands were excised from the gel and were subjected to tryptic digestion and mass spectrometry. Protein and its modification were identified through the database search, and peptide identifications were validated with Peptide Prophet.

Transwell assay

The migration and invasion assays of osteosarcoma cells were performed using 24 wells Boyden chambers. FBS free osteosarcoma cells suspension (U2OS: 5×10^4 /well, U2OS/MTX300 cells: 1×10^5 /well) were placed on the upper layer of the cell culture insert with permeable membrane coated with (invasion) or without (migration) the Matrigel matrix. 10% FBS (migration) or 20% FBS (invasion) cell culture media were added into the lower reservoir, making sure the solution touches the membrane of the insert. The cells in the chambers are then incubation in 37°C (U2OS cells for 12 h, U2OS/MTX300 cells for 24 h). Following incubation, cells left in the upper layer of the insert are wiped off using cotton swab, cells across the inserts are fixed by dipping the insert into 4% paraformaldehyde for 15 min and stained by 1% crystal violet solution for 30 min at room temperature. The inserts are washed with 1 X PBS and leave it dry in the air. Finally, the numbers of cells cross the inserts per field of view are counted under microscope.

Cell viability assay

U2OS, or U2OS/MTX300 cells were seeded in 96-well plates at a density of 6,000 cells per well. Cells were then treated with different concentrations of C646 (0, 2.5, 5, 10, or 20 μM) or SA (0, 1, 5, 10, or 20 μM) for the indicated times, and the cell viability was measured by MTT assay.

***In vitro* acetylation assay**

Rab22a-NeoF1-WT-SFB and Rab22a-NeoF1-K7R-SFB were purified by IP using

Streptavidin Sepharose beads from HEK293T cells transfected with Rab22a-NeoF1-WT-SFB or Rab22a-NeoF1-K7R-SFB plasmids, followed by eluting with 2 mg/ml D-Biotin solution in 4°C for 6 h. Rab22a-NeoF1-WT-SFB and Rab22a-NeoF1-K7R-SFB were incubated with HA-CBP purified from HEK293T cells in HAT buffer (Millipore) in a 30°C shaking incubator for 1 h. The effect of K7 acetylation was determined using anti-K7ac-Rab22a-NeoF1 antibody by Western blotting.

RhoA GTPase activation assay

RhoA GTPase activation assay was performed with RhoA Pull-down Activation Assay Biochem Kit (bead pull-down format) following the direction of the manufacturer's protocol.

***In vivo* studies in mice**

The study is compliant with all relevant ethical regulations regarding animal research. Animal experiments were approved by the Animal Research Committee of Sun Yat-sen University Cancer Center and performed in accordance with established guidelines. U2OS/MTX300-luc cells stably overexpressing Vector, Rab22a-NeoF1 or its K7R mutant were prepared, and nude mice were purchased from Beijing Vital River Laboratory Animal Technology. 1×10^6 cells in PBS with 1% FBS were injected into distal femur, proximal tibia of each nude mouse (10 mice per group). After two months, lung metastases of U2OS/MTX300-luc cells were measured by *in vivo* fluorescent imaging and all mice were sacrificed and lungs with metastasis were harvested, and wet lungs were weighted and lung metastasis nodes were counted. For the treatment of C646, which was dissolved in ddH2O with 7.7% DMSO and 40% PEG300 at daily dose of 10 mg/kg for 14 days, was intraperitoneally injected into mice after the injection of U2OS/MTX300-luc cells for 3 weeks.

Statistical analysis

All experiments were performed at least three times. Data were analyzed with GraphPad Prism v.8 (GraphPad) and SPSS statistics. Student's *t*-test was used to compare the differences between two groups. *p<0.05 was considered as statistically significant while **p<0.01, ***p<0.001, ****p<0.0001 as highly significant.
